# Acute care nurses’ perceptions of electronic health record use: A mixed method study

**DOI:** 10.1002/nop2.157

**Published:** 2018-05-07

**Authors:** Gillian Strudwick, Linda McGillis Hall, Lynn Nagle, Patricia Trbovich

**Affiliations:** ^1^ Centre for Addiction and Mental Health Toronto ON Canada; ^2^ Lawrence S. Bloomberg Faculty of Nursing University of Toronto Toronto ON Canada; ^3^ Institute of Health Policy, Management and Evaluation University of Toronto Toronto ON Canada

**Keywords:** acute care, health services research, information technology, medical nursing, surgical nursing

## Abstract

**Aim:**

The overall aim of this study is to examine nurses’ perceptions of electronic health record use in an acute care hospital setting.

**Design:**

This study uses a sequential mixed methods design in two phases.

**Methods:**

Phase one consists of a survey of Registered Nurses to understand nurses’ perceptions of electronic health record use. Phase two is comprised of focus groups of a subsample from phase one. Data collection occurred from November 2015 ‐ August 2016 and was done in Toronto, Canada.

**Results:**

In phase one, navigation was found to be a predictor of nurses’ perceptions of electronic health record use. In phase two, participants discussed the following five topics: (1) navigation; (2) functionality; (3) organizational standards; (4) documentation workload and (5) issues of system performance and response time. This study has implications for organizations implementing electronic health records, nursing leaders and electronic health record vendors.

## 1 INTRODUCTION

Electronic health records (EHRs) have been implemented in healthcare organizations worldwide (Stone, 2014). These systems are installed so that organizations can achieve several benefits such as enhanced patient safety (Savage, Titus, Manns, & Lee, 2014), better documentation (Dowding, Turley, & Garrido, 2012) and improved quality of care (Plantier et al., 2017). However, previous research has demonstrated that simply implementing an EHR does not mean that healthcare organizations will achieve these intended benefits (Gephart, Carrington, & Finley, 2015; Koppel, Wetterneck, & Telles, 2008; Patterson, Rogers, Chapman, & Render, 2006; Simon, 2007). For benefits of an EHR to be realized, health professionals need to use the technology in a consistent and effective manner (Simon, 2007). Furthermore, health professionals require high levels of technology acceptance if EHR outcomes are to be obtained (Holden & Karsh, 2010). As nurses represent the largest group of health professionals globally (World Health Organization, 2013), their use of EHRs may influence whether anticipated benefits of using the technology are achieved. Thus, it is important to better understand how various barriers and facilitators influence nurses’ use of EHRs. By understanding these factors, interventions and strategies can be identified to better support nurses’ use of the technology.

## 2 BACKGROUND

Several barriers and facilitators to nurses’ use of EHRs have been reported in the literature, many of which can be categorized in relation to: 1) EHR usability; 2) organizational context; and 3) individual nurse characteristics. The first category, EHR usability barriers and facilitators, is comprised of how easy the technology is to use, its functionality, ease of navigation and its impact on workload (Ammenwerth, Ehlers, Hirsch, & Gratl, 2007; Carayon et al., 2011; Carrington & Effken, 2011; Lu, Hsiao, & Chen, 2012; Maillet, Mathieu, & Sicotte, 2015; Saleem et al., 2015; Schenk et al., 2016; Whittaker, Aufdenkamp, & Tinley, 2009; Yontz, Zinn, & Schumacher, 2015). The second category, organizational context, includes: support from leadership, level of training, level of ongoing support and the physical environment (Lu et al., 2012; Maillet et al., 2015; Saleem et al., 2015; Whittaker et al., 2009; Yontz et al., 2015). The individual nurse characteristic category includes: sex, age, nursing unit, years of experience as a nurse, country of nursing education, years of experience using an EHR, previous experience using an EHR and formal informatics training (Ifinedo, 2016; Yontz et al., 2015). Previous research using usability and organizational context variables has typically been conducted with a subset of variables, rather than including them all in a single study (Ammenwerth et al., 2007; Carayon et al., 2011; Yontz et al., 2015). In addition, Ifinedo (2016) has suggested that individual nurse characteristics may act as moderators to the relationships between several barriers and facilitators and nurses’ use of EHRs. In this study, EHR usability and organizational context variables were conceptualized as independent variables and individual nurse characteristics were viewed as potential moderating variables.

### 2.1 Aims

**Figure 1:**
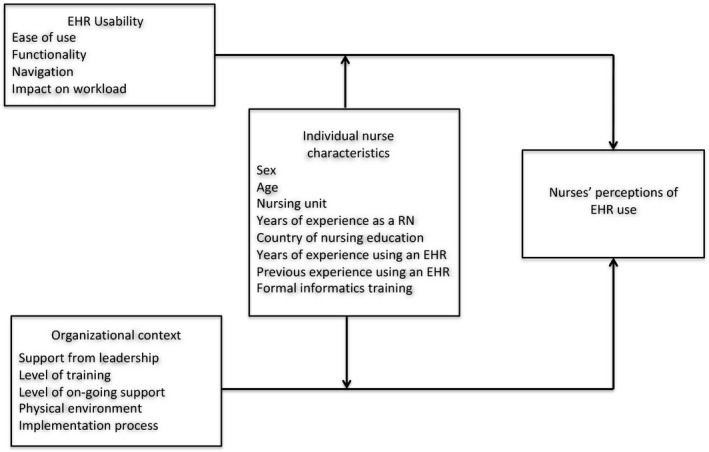
Framework

The overall aim of this study was to examine nurses’ perceptions of electronic health record use in an acute care hospital setting. The specific aims of this study were to: 1) determine if EHR usability variables and organizational context variables are associated with nurses’ perceptions of their EHR use; and 2) examine individual nurse characteristics as possible moderators to these relationships. Figure 1 depicts the relationships between the variables examined in this study.

## 3 DESIGN

This study was conducted using a sequential mixed methods design with both a quantitative and qualitative phase. Specifically, phase one consisted of a quantitative cross‐sectional survey that was administered to nurses using previously validated instruments that had been modified for use in this study. Phase two was qualitative and was comprised of focus groups with a subset of nurses who had participated in phase one.

## 4 METHOD

### 4.1 Setting

The setting for this study was six medical and surgical units in an urban acute care teaching hospital in Toronto, Canada. These clinical units were chosen as they used the same functions of the EHR and have similar processes of care delivery in comparison to other more specialized clinical units such as the emergency department. This organization had an EHR in place for approximately a decade at the time of the study. Functionalities of the EHR included nursing documentation, computerized provider order entry, laboratory results reporting and viewing and an electronic medication administration record. Given the various functions of the EHR present on the study units, the majority of documentation was done electronically with limited use of paper. Nurses at the hospital access the EHR through computers in the nursing station, computers on stands with wheels that can be moved from room to room, as well as computers located in patient rooms. Training related to EHR use at this site occurs when participants are first hired, when a new functionality is implemented, or when a major change is made to the system.

### 4.2 Participants

Participants in both phases of the study consisted of Registered Nurses (RNs) who worked on the medical and surgical units at the study site. The organization did not have other classes of nurses, such as Registered Practical Nurses (RPNs), employed on the included units. To be eligible to participate, RNs were required to have used the EHR for a minimum of 1 year in their regular practice and be employed in their unit for at least 1 year prior to the study initiation. These criteria were selected as it was recognized that it may take nurses time to become familiar and comfortable using the EHR, or working in a new clinical setting (Casey, Fink, Krugman, & Propst, 2004). Nurse managers and other nurses (e.g. nurse educators) were excluded from the study if they did not use the EHR on a regular basis as a part of their practice.

For phase one, recruitment took place through face‐to‐face invitations. The student attended staff meetings and safety huddles on the medical and surgical units at the study site. Envelopes with paper copies of the survey and consent information were provided to potential participants and a drop location on each unit was determined. During phase one, recruitment also took place for phase two. Along with a paper copy of the survey in each envelope, there was an invitation to participate in a focus group later and a focus group response form. Nurses were instructed to fill in the focus group response form with their name and contact information and leave it in the predetermined drop location.

The sample size for this study was determined using Cohen's power analysis for linear regression (Cohen, 1988, 1992), which takes into consideration effect size, the number of independent variables, the level of significance and the study power. With a medium effect size, eight independent variables, a level of significance of 0.05 and a study power of 0.80, it was determined that 130 participants would be required to participate in this study. Given that it was known that not all nurses who were invited to participate in the study would do so, the student planned to ask all of the 329 eligible nurses employed on the study units.

### 4.3 Data collection

The survey was comprised of several instruments used to operationalize the variables included in this study. Specifically, the Workflow Integration Survey (WIS) was used to measure the EHR usability variables ease of use, functionality, navigation and impact on workload (Flanagan et al., 2011). The WIS instrument was developed for use by physicians and nurse practitioners and when used in a previous study had a reported Cronbach's alpha of 0.93 (Flanagan et al., 2011). The instrument consisted of 12 questions and used a 5‐point Likert scale. Organizational context variables were measured using items from the Canada Health Infoway System and Use Assessment Survey (CHISUAS)(Canada Health Infoway, 2015) and items added by the student. Specifically, additional items focused on participants’ perceptions of support from their manager in using the EHR, having enough computers to access the EHR, the location of the computers and the speed of the network connection. The CHISUAS was developed by Canada Health Infoway and is based on the DeLone and McLean Model for Information System Success. The CHISUAS section used in this study consisted of six questions and used a 5‐point Likert scale. Nurses’ perceptions of their EHR use was measured using the Information System Use Instrument which consisted of nine questions and used a 5‐point Likert scale (Abdrbo, Zauszniewski, & Hudak, 2010). This instrument was specifically developed for use with nursing populations. A previous application of the instrument indicated that it had a Cronbach's alpha of 0.82 (Abdrbo et al., 2010). In addition, demographic information (nine items) was collected via a series of questions at the end of the survey.

The survey was pilot tested with five Registered Nurses to assess its clarity, face validity, feasibility and to better understand how long it would take for a participant to complete. Results of the pilot indicated that the survey was easy to understand, clear and took participants approximately 5 min to complete. Changes in the survey included adding a comments section.

The focus group guide (Table 1) was developed based on the findings from phase one and consisted of four main questions, with prompts developed for each question. The first question asked participants about navigation, as results from phase one indicated that navigation was a predictor of nurses’ perceptions of their EHR use. The second question asked participants about functionality given that there were challenges in measuring this variable in phase one. Next, a question was asked each about repetitive charting and how current documentation screens were perceived. These questions were asked based on comments left on surveys by participants in phase one.

**Table 1:** Focus group guide

Question 1: “Navigation” is how logically information is organized in [EHR brand name], and how easily information is located. Can you share with me your experiences “navigating” through [EHR brand name]? Study participants who found [EHR brand name] easier to “navigate” indicated that they used it more. Would the same apply to you?
Question 2: “Functionality” is the extent to which [EHR brand names] has tools or operations available to complete necessary tasks. Participants in this study provided a wide range of comments related to “functionality,” with no specific functionality issue being identified Can you tell me about, or describe your experiences with the “functionalities” of [EHR brand name]?
Question 3: Participants provided a number of comments related to “repetitive” and “double/triple” charting within the [EHR brand name] system. Do any of you want to comment on any experiences you have had of this nature? Have you found this to be the case, and if so, where specifically?
Question 4: There were a number of comments from participants about the documentation and assessment screens in [EHR brand name], and their ability to capture nursing assessments and care provided. Can you tell me about or describe your experiences with the documentation and assessment screens in [EHR brand name]?

### 4.4 Ethics

This study received approval by the study site's Research Ethics Board, in addition to an administrative ethical review at the Health Sciences Research Ethics Board at the University of Toronto. A data transfer agreement was also established between the hospital and the university so that data could be transferred to the university for data analysis. Implied consent was used for the surveys in phase one of this study, whereas in phase two, participants were required to provide written consent for their participation in the focus groups.

### 4.5 Data analysis

Data analysis for phase one was completed using SPSS Version 21. Descriptive statistics were completed to gain a better understanding of the sample. In addition, the internal consistency for each instrument was assessed using Cronbach's alpha. Multivariable linear regression and hierarchical linear regression were used to examine if EHR usability variables and organizational context variables were associated with nurses’ perceptions of their EHR use (Tabachnick & Fiddell, 2013). A chunkwise approach to model building was used to determine which individual nurse characteristics to include as possible moderators (Kleinbaum, Kupper, Nizam, & Rosenberg, 2014). In phase two, the recordings of the focus groups were first transcribed verbatim. The transcriptions were then analysed using a directed content analysis approach using the usability and organizational context variable categories. If no category was appropriate for the participant comment, an “other” category was developed and open (inductive) coding was completed among the remaining comments. To ensure the trustworthiness of the qualitative data analysis, a member of the dissertation committee independently analysed the data in addition to the student (Graneheim & Lundman, 2004). As well, authentic citations have been used in the results section of this manuscript to illustrate the study findings and be transparent with readers (Sandelowski, 1993).

## 5 RESULTS

### 5.1 Phase one

Of the 329 eligible participants, 133 completed the survey in phase one, for a response rate of 40.4%. The mean age of participants was 35.2 (*SD* 9.7) years, with an average of 10.9 (*SD* 8.8) years working as a nurse and an average of 6.8 (*SD* 3.8) years of experience using an EHR. Additional participant characteristics are shown in Table 2.

**Table 2:** Participant characteristics (*N *= 133)

Item	Categories	*N*	%
Sex	Female	121	90.9
	Male	12	9.1
Country of education	Canada	109	82
	Other	9	6.8
	Unknown	15	11.2
Informatics training	Yes	20	15
	No	113	85
Unit	Medical	66	49.6
	Surgical	67	50.4
Experience using another EHR	Yes	47	35.3
	No	83	62.4
	Unknown	3	2.3
Employment status	Full time	106	79.7
	Part time	24	18
	Casual	2	1.5
	Unknown	1	0.8

Cronbach's alphas were calculated for the Workflow Integration Survey and the Information System Use Instrument to assess internal consistency. The Cronbach's alpha for the overall Workflow Integration Survey was acceptable at 0.90. Three of the subscales (ease of use, navigation and impact on workload) also had acceptable Cronbach's alphas of 0.81, 0.78 and 0.81, respectively, however, the Cronbach's alpha for the functionality subscale was 0.55. Given the poor internal consistency of the functionality subscale, the variable could not be included in any subsequent analyses. In addition, the Information System Use Instrument Cronbach's alpha was acceptable at 0.80.

To identify if EHR usability variables were associated with nurses’ perceptions of their EHR use, multivariable linear regression was performed. Assumptions of regression were confirmed, including assessing for multicollinearity, outliers, the presence of a linear relationship between each bivariate, as well as the homoscedasticity, normality and linearity of the residuals. A multivariable model with ease of use, navigation and impact on workload was developed with nurses’ perceptions of their EHR use as the dependent variable. The results indicate that the model explains 13% of the variance in nurses’ perceptions of their EHR use and that navigation was the only significant variable (β = 0.38, *p* = <.01). The other variables (ease of use and impact on workload) were not significant as shown in Table 3.

**Table 3:** Coefficients for the multivariable regression for usability variables predicting nurses’ perceptions of their EHR use

Variable	*R*	*R* ^2^	Adjusted *R* ^2^	*B*	SE *B*	β
Ease of use	0.38	0.15	0.13*	0.15	0.28	0.07
Impact on workload				−0.24	0.20	−0.13
Navigation				0.92	0.25	0.38*

*p = <.01

To assess whether organizational context variables were associated with nurses’ perceptions of their EHR use, multivariable linear regression was also performed. All assumptions of regression were confirmed. A multivariable model with the four organizational context independent variables (support from leadership, level of training, level of ongoing support, physical environment, implementation process) was developed with nurses’ perceptions of their EHR use as the dependent variable. Results of the analysis indicate that the model was not statistically significant (*p* = .51) and therefore organizational context variables may not influence nurses’ perceptions of their EHR use.

Hierarchical linear regression was used to understand if a combination of variables in the framework were associated with nurses’ perceptions of their EHR use. All assumptions of regression were examined and met. In the first block of predictors, years of experience using the EHR and other EHR use, were entered in the model. In the second block, usability variables (ease of use, navigation and impact on workload) were entered. Organizational context variables (support from leadership, level of training, level of ongoing support and physical environment) were entered in the third block. Results of the analysis show that the second block of predictors had a significant *f* change statistic and that the model contributed to 8% of the variance in nurses’ perceptions of their EHR use. Navigation was the only significant predictor variable (β = 0.30; *p *= <.05) as shown in Table 4.

**Table 4:** Hierarchical regression analysis for predictors of nurses’ perceptions of their EHR use

Variable	*R* ^2^	Adjusted *R* ^2^	*R* ^2^ Change	*F* Change	*B*	SE *B*	β
Block 1	0.01	−0.01	0.01	0.66			
Years using EHR					−0.02	0.13	−0.02
Other EHR use					1.20	1.04	0.11
Block 2	0.13	0.08	0.12	4.38*			
Years using EHR					−0.01	0.13	−0.01
Other EHR use					0.89	1.00	0.01
Navigation					0.69*	0.27	0.30
Ease of use					0.29	0.31	0.13
Impact on workload					−0.30	0.23	−0.17
Block 3	0.16	0.08	0.03	0.94			
Years using EHR					0.02	0.13	0.02
Other EHR use					0.70	1.05	0.07
Navigation					0.74*	0.28	0.28
Ease of use					0.31	0.32	0.32
Impact on workload					−0.27	0.23	0.23
Support from leadership					0.23	0.64	0.64
Level of training					−1.30	0.76	0.76
Level of ongoing support					0.73	0.76	0.76
Physical environment					0.08	0.67	0.67

*p = <.05

A chunkwise approach to model building was used to identify if individual nurse characteristics were possible moderators to the relationships between the usability and organizational context variables and nurses’ perceptions of their EHR use. The “chunkwise” approach is a method for determining which individual nurse characteristics to include in models examining the relationships between the independent and dependent variables of interest, by reviewing the *f* change statistic and its significance when possible moderators are added to a model (Kleinbaum et al., 2014). Age, years of experience using an EHR and other EHR uses were identified as individual nurse characteristics with both theoretical significance and enough variability in the participant responses to be included as possible moderators in the analyses. Similar to multivariable linear regression, all models were assessed for the assumptions of regression and met these criteria. When the chunkwise model building was conducted, none of the models demonstrated a significant *f* change statistic when individual nurse characteristics were added. Results of this test therefore suggest that individual nurse characteristics are not moderators to the relationships between the usability and organizational context variables and nurses’ perceptions of their EHR use.

### 5.2 Phase two

In phase two, focus groups were conducted with a total of six participants. Three nurses were present during each focus group. Issues related to: (1) navigation; (2) functionality; (3) organizational standards; (4) documentation workload and (5) issues of system performance and response time, were identified by participants (Table 5).

**Table 5:** Summary of phase two results

Issue	Description	Example
Navigation	Nurses reported that it was difficult to document assessments and care given that there were multiple places within the record to document information. Nurses also described that to find information they would have to open and close each screen to find what they were looking for, and that this was both tedious and time consuming.	“There's like wounds skin integrity, and they ask is there anything abnormal, where is it, the location, but then you have to do documentation of their wound dressing change, it's there again. It's like why are you, again, why are you doing it twice, in a way? And who's looking at which one? What, what one's actually… people are actually looking to? Are we just documenting to document, or is it actually of need or kind of like of use?” (Focus Group 1, Participant 1)
Functionality	Nurses described functions of the EHR that were particularly useful including: the ability to communicate with pharmacy, access to calculators and educational materials related to drugs and clinical information, the clinical documentation screens and referral forms. Participants also described how they liked functions of the record that allowed them to see trends in data over a period of time, and they also described finding interoperability with other medical devices to be useful.	“One thing we had asked for is…when a new order or suggest order comes, like when you first open that chart, it pops up. We asked because a lot of times we'll get stat orders and no one calls us to tell us and if you haven't checked it for a while, then you don't know, it's like been a couple of hours…Because, then the doctors complain that …the stat order wasn't given right when they ordered it and somebody didn't call me and it's like back and forth.” (Focus Group 2, Participant 1)
Organizational standards	Given that there are multiple places to document the same information within the record, nurses wanted clarity with regard to what and where to document patient data.	“…there's so many options to put things… there's no standard of where to put the information…”(Focus Group 1, Participant 1)
Documentation workload	Focus group participants described how addressing the ambiguity nurses’ felt with where and what to document, might support them in reducing documentation workload. The addition of documentation forms over the lifespan of the EHR may have contributed to an increasing workload for nurses.	“It just seems to me … that every year there's more expected to chart from nurses, like, they add in, like, confusion assessment, but that wasn't there…5 years ago. There's, um, like things that are, I would… they are important, but like, it just seems like okay you have to do, like, five different [EHR brand name] things in the first year and then next year they come up with, okay, you have to do these two more assessments in addition to your charting and the next year after that, oh, another assessment that they add to [EHR brand name]… And it just seems… it will get overwhelming or it is already overwhelming the amount of stuff that we have to chart” (Focus Group 2, Participant 3)
Issues of system performance and response time	Nurses described issues with system performance and response time, particularly when certain forms were being used to document.	“Freezes for, like, a good 10 s, because there's just so much information that it loads up and then you only, like, for sometimes if you're charting on a wound, you're only charting, like, to small portion of that” (Focus Group 2, Participant 3)

## 6 DISCUSSION

The results of this study show that nurses experience challenges navigating through the EHR that influence how they perceive their use of it. Other EHR usability variables (ease of use, functionality and impact on workload), organizational context variables (support from leadership, level of training, level of ongoing support and physical environment) and individual nurse characteristics (years of experience using an EHR, other EHR use, age) were not significantly associated with nurses’ perceptions of their EHR use in the quantitative phase of this study. However, several the variables were described by nurses in the focus groups during phase two and thus findings from both phases of this study are discussed below.

### 6.1 EHR usability variables

The broader system ease of use challenges identified through the focus groups in this study are congruent with the findings of other studies with health professional participants (Garavand et al., 2016; Harrington, 2015; Lowry et al., 2014; Staggers, Kobus, & Brown, 2007). This implies that currently available EHRs have not been adequately designed to support health professionals in using the various functions of the systems. Unfortunately, design‐related changes are best addressed pre‐market when the systems have yet to be implemented in healthcare organizations. Once an EHR is in place it is difficult to make any significant design changes that would influence the ease of use of the system experienced by nurses.

One way that nurses in this study were able to adapt to some of the ease of use challenges was to create workarounds. Workarounds are ways that nurses interact with the EHR that are unintended by the vendor or by the organization, but better support the experiences that nurses have using it. An example of a workaround identified in this study was that nurses would login to a patient's record on two separate computers so that they could view different parts of the record at the same time. The way the EHR system was designed in the study organization allowed users to access one section of the record at a time, however, nurses indicated that there were times when accessing multiple sections was required. The presence of this workaround indicates that the design of the EHR is not supportive of end user practice (Debono et al., 2013). Numerous studies have examined workarounds and have shown that although the workarounds may improve the user experience for the nurse, they may be created at the expense of something else, for example, patient safety (Carrington & Effken, 2011; Debono et al., 2013; Edwards, Moloney, Jacko, & Sainfort, 2008; Koppel et al., 2008; Schoville, 2009).

With regard to functionality, although the variable was not examined quantitatively in this study, focus group participants described several functions of the EHR that supported nursing practice. These functions (e.g. communicating with pharmacy) were described as having a positive influence over their use of the technology. The results, therefore, suggest that having functions of the EHR that support nurses’ work may enhance nurses’ use of the technology. An implication of this finding to organizations implementing EHRs is to ensure that there is an adequate representation from nurses during the requirement gathering and selection phases of the procurement of new technology. Getting this right translates into an EHR design that supports nursing practice.

One of the specific functions suggested by nurses in this study was for alerts to be created in the EHR when new orders are entered. Results of the research on the use of alerts in clinical settings are mixed. On one hand, studies have shown that when alerts are used in specific scenarios, such as letting a clinician know about immunization requirements (Fik, Grundmeier, Biggs, Localio, & Alessandrini, 2007), or to remind them to complete a specific screening (Schnall et al., 2010), there may be benefits. Alternatively, having too many alerts may lead to “alert fatigue”. With alert fatigue, nurses may inadvertently ignore the alerts due to the volume of alerts occurring on a daily basis. Given the number of orders that might be expected on a medical and surgical unit, it may be difficult to implement an alert system for all new orders. Instead, alerts could be considered for orders that are “urgent” or “stat” only, or a different mechanism for alerts could be considered such as a whiteboard.

In this study, navigation was significantly related to nurses’ perceptions of their EHR use in phase one. This finding indicates that EHRs that are difficult for nurses to navigate, negatively influence their use of the system. One of the implications of this finding for healthcare organizations is the importance of conducting a navigational assessment when either selecting a new system, or when making any changes to the system currently in place. As a result, healthcare organizations will be able to able to better understand whether the selected system or design change will adequately support the largest user group. Selecting and/or designing a system that is easy to navigate allows for the effective use of the various system functions that can be of value to nurses.

Results of this study related to navigation are in alignment with those in previous research. For example, it has been shown that when health professionals have a difficult time finding information in an EHR due to poor navigation, their use of the EHR to complete tasks is decreased (Christensen & Grimsmo, 2008). A study of medical students in the United States showed that poor EHR system navigation contributed to students not being able to find critical patient information (Yudkowsky, Galanter, & Jackson, 2010). As well, a study of nurses in two community care settings showed that nurses were not able to maximally use the EHR due to poor system navigation (Sockolow, Liao, Chittams, & Bowles, 2012). Nurses in the community managed this challenge by spending time before each patient visit navigating through the record and reviewing it. Interestingly, a separate study reviewing the search queries in an EHR identified that navigational related searches made up 14.5% of all queries (Natarajan, Stein, Jain, & Elhadad, 2010). This reflects one of the ways that health professionals have learned to overcome EHR navigational challenges.

In the focus groups, nurses described challenges with their workload related to EHR use, despite this variable not being significant in the quantitative analysis. Nurses described how they were routinely staying beyond their shift to complete necessary documentation. They identified that the added workload might be related to the system design (e.g. having multiple places to document the same information) and the lack of clear organizational expectations for use of the EHR. Healthcare organizations implementing these systems will want to ensure that there is a streamlined approach to documentation such that information can be documented efficiently with minimal requirements for duplicate documentation. This also means reviewing existing documentation when new forms are added to the EHR to avoid potential duplication of effort. In addition, having clear organizational expectations for nursing documentation communicated during EHR training sessions and clinical orientation and reflected in organizational policies and procedures may be warranted.

The finding of having documentation workload challenges aligns with studies done with both nurses and other health professionals (Bae & Encinosa, 2016; Poissant, Pereira, Tamblyn, & Kawasumi, 2005; Stokowski, 2013). However, the present study adds insights to the potential link between system design, organizational expectations and nurses’ documentation workload. Future research directed at examining these relationships may be of value.

### 6.2 Organizational context variables

Organizational context variables were not significant in any of the quantitative analyses but discussed in the focus groups by nurses as potential influencers of their EHR use. For example, issues of system performance and response were discussed as negatively influencing nurses’ perceived use of the EHR. Organizations will need to ensure that the appropriate technical infrastructure (e.g. number, type, location of devices) is in place so that system performance (e.g. application and network response) is not impeded.

Using a single site may have contributed to the lack of variability in participant responses. Nurses at the study site receive the same EHR training as one another regardless of their clinical unit. They also had the same organizational supports available (e.g. help desk), the same EHR system, a similar unit layout/physical environment, similar ways to access the system and a unit manager reporting to the same director as the other unit managers. It is also possible that organizational context variables may have had an influence on nurses’ use of the EHR when it was first implemented; however, since the system has been in place for approximately a decade, this effect may no longer be present.

### 6.3 Individual nurse characteristics

Although individual nurse characteristics in this study were not significantly related to nurses’ perceptions of their EHR use, it does not mean that these variables are not meaningful during earlier stages in the adoption of the technology. It may be that the effect of these variables was not present at the time when the study was conducted, given that the participants had already been using the EHR for several years. However, if the study had been done when the EHR was first implemented, it is possible that individual nurse characteristics may have influenced nurses’ perceptions of their EHR use. Over time these effects may have worn off. In the future, a longitudinal study may be of value to examine this hypothesis.

### 6.4 Limitations

This study had several limitations that should be considered in the context of the results presented. The study was done at a single site with a unique organizational context and a commercially available EHR. The generalizability of the study results to other settings, organizations and those using different EHR systems is unknown. In addition, the functionality variable demonstrated poor internal consistency in how it was measured; as a result, it could not be included in any subsequent statistical analyses. Therefore, it is unknown what influence the functionality variable may have had on the survey results. It should be noted that nurses in the focus groups were asked to discuss the functionality variable and the results of these discussions indicated that the functionality of the EHR may influence nurses’ use of it. However, these discussions should be interpreted with caution given the small size of the focus groups. Despite reaching data saturation as indicated through the repetition of similar topics and themes, it is possible that there is additional information that was not communicated during the focus groups that would allow for a better understanding of nurses’ use of the EHR.

## 7 CONCLUSION

This study has shown that nurses at the study site experienced challenges using the EHR, particularly those related to navigation, functionality, organizational standards, documentation workload and system performance and response time. Healthcare organizations may be able to better support nurses’ use of these systems by ensuring that nurses are involved in the EHR procurement process (or design change process), having clear expectations and standards for use, eliminating areas in the record that require duplicate documentation and ensuring that the proper technical infrastructure is in place to support adequate system performance. Ensuring that practicing nurses are involved in the design, procurement and implementation of EHRs may support enhanced use. Future research that examines factors that influence nurses’ perceptions of their EHR use longitudinally should be considered.

## CONFLICT OF INTEREST

No conflict of interest has been declared by the authors.
